# Programmed Cell Senescence in the Mouse Developing Spinal Cord and Notochord

**DOI:** 10.3389/fcell.2021.587096

**Published:** 2021-01-26

**Authors:** Jorge Antolio Domínguez-Bautista, Pilar Sarah Acevo-Rodríguez, Susana Castro-Obregón

**Affiliations:** División de Neurociencias, Instituto de Fisiología Celular, UNAM, Mexico City, Mexico

**Keywords:** Cdkn1a/p21^CIP1/WAF^, Cdkn2a/p16^INK4A^, spinal cord, notochord, endothelial cells, motoneurons, mouse development

## Abstract

Programmed cell senescence is a cellular process that seems to contribute to embryo development, in addition to cell proliferation, migration, differentiation and programmed cell death, and has been observed in evolutionary distant organisms such as mammals, amphibians, birds and fish. Programmed cell senescence is a phenotype similar to stress-induced cellular senescence, characterized by the expression of the cell cycle inhibitors p21^CIP1/WAF^ and p16^INK4A^, increased activity of a lysosomal enzyme with beta-galactosidase activity (coined senescence-associated beta-galactosidase) and secretion of growth factors, interleukins, chemokines, metalloproteases, etc., collectively known as a senescent-associated secretory phenotype that instructs surrounding tissue. How wide is the distribution of programmed cell senescence during mouse development and its specific mechanisms to shape the embryo are still poorly understood. Here, we investigated whether markers of programmed cell senescence are found in the developing mouse spinal cord and notochord. We found discrete areas and developmental windows with high senescence-associated beta galactosidase in both spinal cord and notochord, which was reduced in mice embryos developed *ex-utero* in the presence of the senolytic ABT-263. Expression of p21^CIP1/WAF^ was documented in epithelial cells of the spinal cord and the notochord, while p16^INK4A^ was observed in motoneurons. Treatment with the senolytic ABT-263 decreased the number of motoneurons, supporting their senescent phenotype. Our data suggest that a subpopulation of motoneurons in the developing spinal cord, as well as some notochord cells undergo programmed cell senescence.

## Introduction

Embryonic development is achieved by strongly coordinated mechanisms of cell migration, proliferation, differentiation and programmed cell death. Recently, programmed cell senescence was identified as an additional process that controls mouse development (Munoz-Espin et al., 2013; Storer et al., 2013). The presence of programmed cell senescence has also been reported during specific structures and windows of amphibian, chick and fish development, suggesting that programmed cell senescence contributes to vertebrate organogenesis and may have arisen in evolution as a developmental mechanism (Storer et al., 2013; Davaapil et al., 2017; Da Silva-Alvarez et al., 2020).

The development of the spinal cord and the differentiation of motoneurons are intensively studied due to its importance in organismal physiology and as in search for therapeutic targets for diseases such as amyotrophic lateral sclerosis. During the development of the spinal cord, the roof plate and the floor plate, located in the dorsal and ventral regions of the neural tube, regulate the dorsoventral patterning of different neuron populations by expressing WNT and BMP proteins dorsally, and SHH ventrally (Chizhikov and Millen, 2004; Placzek and Briscoe, 2005). Interestingly, discrete populations of floor plate cells have been identified along the anteroposterior axis, suggesting a variable mode of floor plate induction (Placzek and Briscoe, 2005). Adjacent to the ventral neural tube is located the notochord, a rod-like mesodermal structure that runs the anterior-posterior length, which becomes the rostrocaudal axis. The notochord is a source of developmental signals, such as the Hedgehog proteins, that play key roles in the patterning and proliferation of several organs (Corallo et al., 2015). As a result of signaling gradient of molecules secreted from the notochord and the floor plate, different progenitor cells located in the dorso-ventral axis of the neural tube give rise to V0-3 interneurons and motoneurons (Davis-Dusenbery et al., 2014).

In the developing spinal cord, blood vessels sprout from the perineural vascular plexus and invade the spinal cord at the ventral side. The motoneurons play an active role during blood vessel formation in the spinal cord by expressing vascular endothelial growth factor (VEGF) to allow blood vessel growth, but at the same time express a soluble VEGF receptor to titrate the availability of the growth factor in order to pattern the vasculature and block premature ingression of vessels by an attraction-repulsion mechanism (Himmels et al., 2017).

During the development of the spinal cord, motoneurons are produced in excess and compete for supply of trophic factors produced by synaptic targets. Approximately half of the initially produced motoneurons undergo programmed cell death due to lack of trophic support (Oppenheim, 1989). Programmed cell death occurs mainly by apoptosis in the developing embryo; however, while the lack of caspase-3 or caspase-9 results in obvious and severe forebrain malformations, the organization and morphology of the spinal cord appears normal at embryonic and postnatal stages (Oppenheim et al., 2001). Such lack of an obvious phenotype in caspase-3 or caspase-9 knockout mice may be due to a compensation mechanism of remaining caspases (Buss and Oppenheim, 2004), although it is also possible that genetic elimination of apoptosis is masked by alterative mechanisms to eliminate cells such as autophagic cell death and programmed senescence. In fact, dying neurons in caspase-3 and caspase-9 deficient embryos exhibit a different morphology with cell shrinkage, increased cytoplasmic and nuclear density, and very little chromatin condensation(Oppenheim et al., 2001). A former work showed that reactive oxidative species signal to induce motoneuronal cell elimination by a caspase-independent mechanism, as the number of motoneurons was higher in developing spinal cords explants cultured in the presence of a superoxidase dismutase-catalase mimetic than with caspase inhibitors (Sanchez-Carbente et al., 2005).

While autophagy is primarily a survival mechanism frequently activated to eliminate damaged proteins and organelles, evidence shows that autophagy contributes to programmed cell death for example during the *Drosophila* larval salivary glands regression (Tracy and Baehrecke, 2013), and the elimination of the interdigital web during limb development (Arakawa et al., 2017). Since the number of motoneurons increased in spinal cord explants cultured in the presence of the PI3K inhibitor LY294002, it was suggested that autophagy contributes to eliminate supernumerary motoneurons during spinal cord development, in a mechanism that occurs without DNA fragmentation (i.e., TUNEL negative) (Sanchez-Carbente et al., 2005). Therefore, alternative mechanisms occur simultaneously to adjust the number of motoneurons during spinal cord development, and programmed cell senescence could be one of them.

Cellular senescence is characterized by a durable exit from the cell cycle and acquisition of a flattened morphology; expression of the cell cycle inhibitors p16^INK4A^ (coded by *Cdkn2a* gen), p21^CIP1/WAF^ (coded by *Cdkn1a* gen), and/or p53; expansion of mitochondrial and lysosomal networks; and a high level of senescence-associated-β-galactosidase activity (SA-β-gal). Senescent cells are metabolically active and influence the tissue microenvironment through their secretory phenotype (chemokines, cytokines, growth factors, metalloproteases, etc.) (Czarkwiani and Yun, 2018). During development, programmed cell senescence contributes to tissue remodeling in structures such as the mesonephros, endolymphatic sac and apical ectodermal ridge in mice, but it still has to be understood how they help to shape the embryo. Programmed cell senescence could cooperate with programmed cell death to eliminate transient structures and adjust the final number of cells, and could also secrete developmental signals. Mechanistically, programmed cell senescence depends on the p21^CIP1/WAF^ cell cycle inhibitor and is regulated by the TGF-β/SMAD, PI3K/FOXO and ERK1/2 pathways (Munoz-Espin et al., 2013; Storer et al., 2013). In amphibians also TGF-β triggers programmed cell senescence, although it is independent of ERK1/2 pathways (Davaapil et al., 2017). In contrast to other non-proliferating states as senescence or terminal differentiation, quiescence is a reversible cell cycle exit that takes place in cells that require a strict proliferation regime, such as stem cells. Molecularly, the transcriptional repressor HES1 safeguards against irreversible cell cycle exit during cellular quiescence, and prevents senescence (Sang et al., 2008; Sueda et al., 2019).

Senescent cells activate several prosurvival pathways and become resistant to extrinsic and intrinsic proapoptotic stimuli (Kang, 2019). Members of the BCL-2 family, specifically antiapoptotic BCL-X_L_ and BCL-W, are essential for the survival of senescent cells (Hernandez-Segura et al., 2018). Thus, an approach to eliminate senescent cells for therapeutic or experimental purposes involves compounds, referred to as senolytics, that can specifically induce senescent cells to die. Compounds discovered so far include ABT-263 (Navitoclax), which inhibits different members of the BCL-2 family of antiapoptotic proteins (Chang et al., 2016; Yosef et al., 2016; Hernandez-Segura et al., 2018). This senolitic has been used to demonstrate the presence of programmed cell senescence in zebrafish (Da Silva-Alvarez et al., 2020).

Here we investigated whether there are cells undergoing programmed senescence during mammal spinal cord and notochord development. We found in the roof plate, floor plate, motoneuron zone, and notochord, cells with high SA-β-gal activity. Several cells in the notochord and endothelial cells in the spinal cord also expressed the tumor suppressor p21^CIP1/WAF^; we did not find motoneurons expressing p21^CIP1/WAF^, but we did observe that a subpopulation of motoneurons express p16^INK4A^ and not the quiescent marker HES1. Supporting their senescent nature, a subpopulation of motoneurons was eliminated by the senolytic drug ABT-263. It is tempting to speculate that programmed cell senescence cooperate with programmed cell death to regulate the motoneuronal population during spinal cord development.

## Materials and Methods

### Timed Mating and Open-Book Preparations of Spinal Cords

Mice used in the present study were handled and cared according to the animal care and ethics legislation. All procedures were approved by the Internal Committee of Care and Use of Laboratory Animals of the Institute of Cell Physiology- UNAM (IFC-SCO51-18). Mice had *ad libitum* access to water and food. Estrous cycle of female CD1 mice was monitored by vaginal lavage examination. Females in proestrus or estrus were placed in cages with fertile males overnight. The following morning (9:00 a.m.) was considered as embryonic day 0.5 (E0.5) if a vaginal plug was found. On days E10.5 to E14.5, dams were killed by cervical dislocation, and the uterus was washed in PBS. Embryos were dissected under a stereoscopic microscope. For open-book preparations, spinal cords were dissected from E12.5 to E14.5 embryos, opened dorsally and freed from meninges.

### SA-β-Gal Assay

SA-β-gal assay was performed as described elsewhere (Debacq-Chainiaux et al., 2009). Whole embryos from stages E10.5 and E11.5, or open book preparations from spinal cords from E12.5 to E14.5 were washed twice in PBS and fixed 5 min with 2% formaldehyde plus 0.2% glutaraldehyde in PBS at room temperature. Reaction was performed by incubating embryos at 37°C overnight in reaction solution containing 1 mg/ml X-gal, 40 mM citric acid/sodium phosphate buffer pH 6.0, 5 mM potassium ferrocyanide, 5 mM potassium ferricyanide, 150 mM sodium chloride, and 2 mM Magnesium chloride. After SA-β-gal assay, spinal cords were fixed with 4% paraformaldehyde for 20 min. Some samples were used to perform immunofluorescence after fixation with 4% paraformaldehyde.

### Immunofluorescence

Whole embryos or dissected spinal cords were fixed in PBS containing 4% paraformaldehyde for 30 min at room temperature, washed twice with PBS, and cryoprotected overnight with 30% sucrose in PBS. Embryos were cryosectioned by embedding in Tissue-Tek^®^ O.C.T., histological sections of 40 μm were obtained using a Leica Cryostat and mounted on Superfrost plus slides. Sections on slides were washed three times with PBS for 5 min each time followed by permeabilization with 0.5% Triton^TM^ X-100 in PBS for 30 min at room temperature. Afterwards, tissue sections were blocked with 2% Bovine Serum Albumin in PBS for 1 h and then incubated overnight at 4°C with the following antibodies: ISLET-1 (1:200, abcam, ab109517), HES1 (1:200, Santa Cruz, sc-25392), F4/80 (1:200, abcam, ab6640), BCL-X_L_ (1:50, Santa Cruz, sc-1690), PECAM (1:50, BD Pharmingen, 550274), ISLET-1 (1:25, DSHB, 40.2D6), Ki67 (1:500, abcam, ab15580), and p21^CIP1/WAF^ (1:100, abcam, ab109199). Next day, secondary antibodies Alexa Fluor^®^ 488 anti-rat (Invitrogen, A21208), Alexa Fluor^®^ 488 anti-mouse (Invitrogen, A11029), Alexa Fluor^®^ 488 anti-rabbit (Invitrogen, A11034), Alexa Fluor^®^ 594 anti-rabbit (Invitrogen, A11037 Alexa Fluor^®^ 647 anti-rabbit (Jackson Immuno Research, 711-606-152) or Alexa Fluor^®^ 546 anti-rat (Invitrogen, A11081) were incubated at a dilution of 1:500 in 2% BSA for 1 h at room temperature. For p16^INK4A^ (1:150, Santa Cruz, sc-1661) antigen retrieval with citrates buffer was performed before permeabilization/blocking step with 0.5%Tween/5%BSA and 0.1% H_2_O_2_. Slides were mounted with Fluoromount-G^TM^ (Electron Microscopy Sciences, 1798425), and images were collected in a LSM800 (Zeiss) confocal microscope. All images were collected with 1 Airy Unit aperture of pinhole. The acquisition of SA-β-gal images by confocal microscopy was performed as described elsewhere (Levitsky et al., 2013). The primary and secondary antibodies were incubated in presence of detergents, 0.1% Tween/0.25%Tritón and 0.1%Tween, respectively. For quantification of motoneurons, embryos were cultured *ex-utero* as described below and processed for immunofluorescence using the motoneuron marker ISLET-1. Confocal microscopy images of cervical cross-sections were collected using the EC Plan-Neofluar 20x/0.50 M27 or Plan-Apochromat 40x/1.3 Oil DIC (UV) VIS-IR M27 objectives. CZI files were opened in the Fiji software, and ISLET-1^+^-cells in the motoneuron zone were manually counted using the Multi-point tool. A single confocal plane per embryo was used for ISLET-1^+^-cell quantification. Data was quantified was analyzed as indicated in the statistical analysis section.

### DNA-Damage Induced Senescence in Mouse Embryonic Fibroblasts and Live Dead Assay

Mouse embryonic fibroblasts (MEFs) from passages between 3 and 5 were plated at a density of 6,750 cells/cm^2^ in DMEM supplemented with 10% fetal bovine serum plus antibiotic/antimycotic (Life Technologies, 15240062). Next day, aqueous solution of etoposide was added to a final concentration of 120 μM for 2 h. Then, the medium was replaced with etoposide-free DMEM plus fetal bovine serum. Cells were cultured for 5 d at 37°C and 5% CO_2_ without changing the medium. Then, cells were treated for 20 h with 10 μM ABT-263 or equivalent concentrations of DMSO (0.2%) as vehicle-control. After ABT-263 treatment cell viability was quantified using the LIVE/DEAD™ Viability/Cytotoxicity Kit (ThermoFischer, L3224). Briefly medium was replaced with serum-free medium containing 1 μM calcein and 1 μM ethidium homodimer for 25 min. Then, epifluorescent images were collected in live cells using 50% glycerol in PBS as mounting medium. Live or dead cells were quantified manually using the multi-point tool from Fiji software.

### Embryo Culture

Mouse embryos of E12 stage were cultured in a roller bottle system using reported protocols (Takahashi and Osumi, 2010; Kalaskar and Lauderdale, 2014). Uterus from pregnant mice were dissected, washed twice in sterile PBS at 37°C, and transferred to DMEM/F12 under a sterility hood. Embryos surrounded by the intact yolk sac were detached from the placenta and then freed from the yolk sac without separating it from the embryo. Dissected embryos were transferred to culture medium at 37°C consisting of KnockOut DMEM (Life Technologies, 10829018) containing 10% KnockOut Serum Replacement (Life Technologies, 10828028), N-2 supplement (Life Technologies, 17502048), 2% bovine serum albumin suitable for cell culture (RMBIO BSA-BAF-25G), and antibiotic/antimycotic (Life Technologies, 15240062). Embryos were pre-cultured in rolling bottles in an incubator at 37°C with continuous flow of 95% O_2_/5% CO_2_. Then, good heart rate and blood circulation was verified under a stereoscopic microscope for culturing the embryos for additional 15 h in the presence of the autophagy inhibitor spautin-1 (SIGMA, SML0440) or the senolytic ABT-263 (Chemgood, C-1009). Equivalent concentrations of DMSO were included as control conditions to exclude vehicle effect.

### Western Blot

As LC3-II is not detectable in lysates of embryonic spinal cord by Western blot, it was enriched by preventing its degradation with chloroquine, added to culture medium to a final concentration of 100 μM during the last hour of embryo culture. The spinal cords were dissected with meninges and total lysates were obtained using a buffer consisting of 68.5 mM Tris HCl at pH 6.8, 2% SDS, and protease inhibitors cOmplete™ULTRA tablets (Sigma 5892791001). Lysate was cleared using a pestle sonicator for 10 s at 30% cycle, and centrifuging at 10,000 g for 10 min at room temperature. Protein was quantified using the DC^TM^-protein assay kit (Bio-Rad, 5000112) using bovine serum albumin as standard. Protein was subjected to electrophoresis, transferred to a PVDF-FL membrane and analyzed using the LC3 antibody (MBL, PD014 at 1:2000 dilution overnight at 4°C) or anti-β-actin (Santa Cruz, sc-47778 at 1:10,000 for 1 h at room temperature). Secondary antibodies (IRDye^®^ 800CW Goat anti-Rabbit (LI-COR, 92632211)and IRDye^®^ 680RD Goat anti-Mouse IgG (LI-COR, 92568070) were diluted 1:10,000 and incubated for 1 h at room temperature. Bands were visualized in an Odyssey^®^ CLx Imager, and quantified with the Image Studio Lite Version 5.2 software.

### Statistical Analysis

Data are plotted as mean ± standard error of the mean. One-way ANOVA followed by Bonferroni's multiple comparisons test was performed using GraphPad Prism version 6.01 for Windows, GraphPad Software. *P ≤ 0.05, **P ≤ 0.01, ***P ≤ 0.001.

## Results

### SA-β-gal Activity Is Observed in Motoneurons and Phagocytic Cells of the Developing Spinal Cord

**Figure 1:**
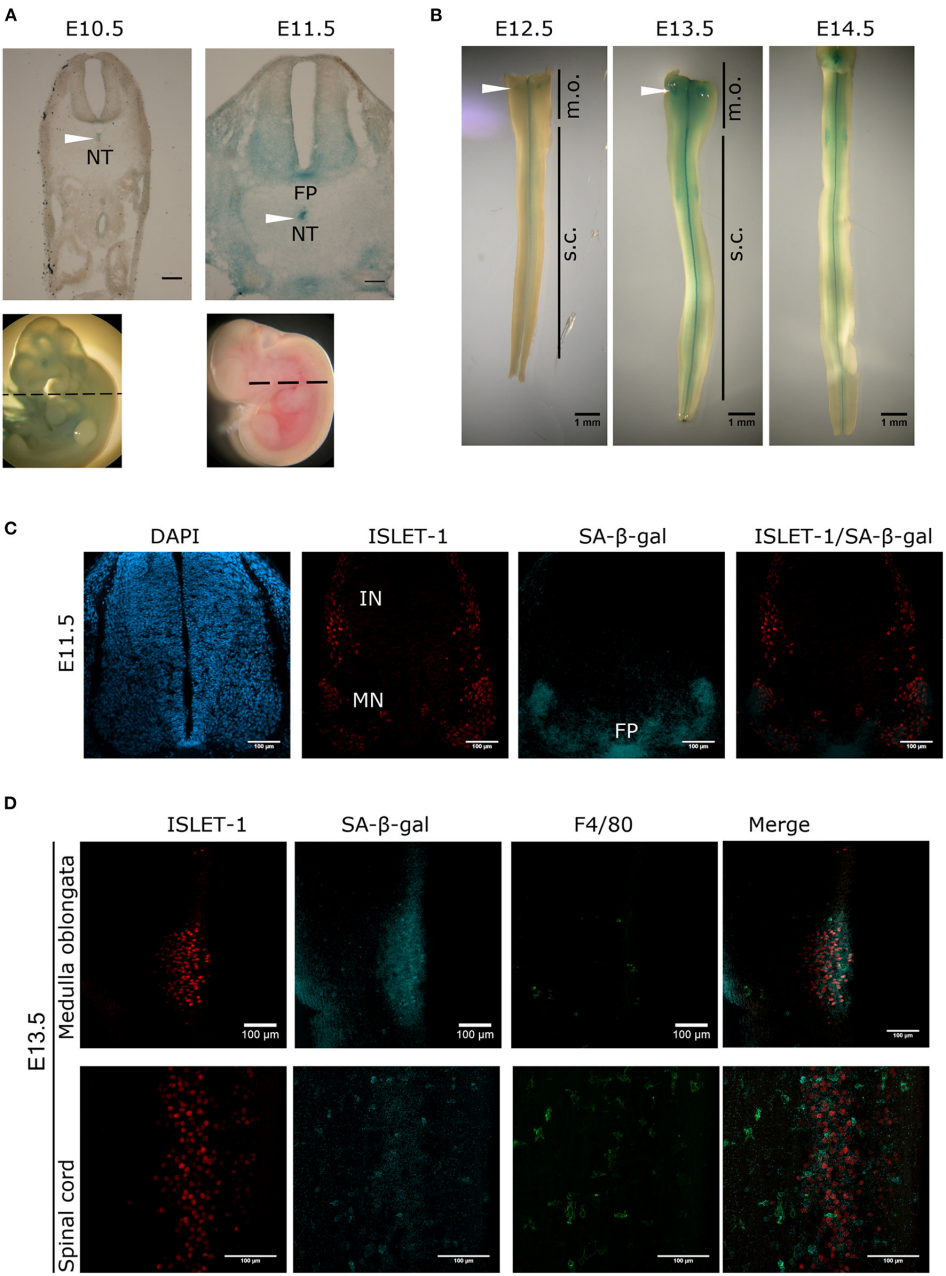
Transient and region-specific SA-β-gal activity in the mouse embryonic spinal cord and notochord. **(A)** Whole embryos from E10.5 and E11.5 were stained for SA-β-gal activity and cross-sectioned at the position indicated with dotted line in embryos shown in the lower row. Notice that the notochord (NT, arrowheads), the floor plate (FP) and the ventral neural tube where motoneurons develop have high SA-β-gal activity at E11.5. Scale bars represent 100 μm. **(B)** Open-book preparations of spinal cords from E12.5 to E14.5 were subjected to SA-β-gal activity assay. SA-β-gal activity is strongly detected in the floor plate and at lower extent in the region corresponding to motoneurons of the spinal cord (s.c.). Arrowheads indicate SA-β-gal positive cells in the medulla oblongata (m.o.). **(C)** Whole E11.5 embryos were stained for SA-β-gal activity, thoracic sections were obtained and processed for immunodetection of ISLET-1. Confocal analysis shows that SA-β-gal activity overlaps with the motoneuron (MN) zone and the floor (FP) plate, while interneuron (IN) cells that also express ISLET-1, do not display SA-β-gal activity. Maximal projections are shown. Scale bars represent 100 μm. **(D)** Open-book spinal cord of stage E13.5 were stained for SA-β-gal activity and then subjected to whole-mount immunofluorescence against ISLET-1 and F4/80. Cells positive for ISLET-1 in the medulla oblongata (upper row) correspond to cells indicated with white arrowhead in **(B)**. Detection of SA-β-gal activity by confocal microscopy combined with immunodetection of motoneurons (ISLET-1) and activated macrophages (F4/80) shows that these two cell types display SA-β-gal activity both in the medulla oblongata (upper row) and spinal cord (lower row). Maximal projections are shown. Scale bars represent 100 μm. Representative images are shown from at least three embryos or dissected spinal cords analyzed per immunofluorescence.

To search for the presence of programmed cell senescence in the mouse developing spinal cord, colorimetric detection of SA-β-gal was performed. Cross-sections of E10.5 and E11.5 embryos showed a dynamic SA-β-gal activity. While at E10.5 no signal was detectable in the neural tube, and only a faint signal was detected in the notochord, at E11.5 a strong signal was observed in the notochord; accordingly, SA-β-gal activity has also been observed in the chicken notochord (Lorda-Diez et al., 2015). We also observed SA-β-gal activity in the floor plate and the ventral area of the neural tube, where the motoneurons are located (Figure 1A). We then analyzed the pattern of SA-β-gal activity in open-book preparations of spinal cords from E12.5 to E14.5 embryos. Interestingly, the enzymatic activity observed in the floor plate and motoneuron zone gradually decreased from the rostral region toward the caudal region as development advanced (Figure 1B). Note that in the medulla oblongata there is a cluster of cells positive for SA-β-gal activity (arrowheads, Figure 1B).

To demonstrate that the staining of SA-β-gal overlaps with the motoneuron zone, the motoneuron marker ISLET-1 was immunodetected in cross-sections of E11.5 embryos previously stained for SA-β-gal and analyzed by confocal microscopy. As expected, the SA-β-gal activity overlaps with the motoneuron columns, but not with the interneurons, which also express ISLET-1. Importantly, SA-β-gal activity was confirmed in the floor plate (Figure 1C). Interestingly, in open-book preparations of E13.5 spinal cords we observed that the SA-β-gal in the medulla oblongata also overlaps with motoneurons located there (Figure 1D, upper row), as well as in the spinal cord (Figure 1D, lower row). Since macrophages have high lysosomal activity (Hall et al., 2017), it could be possible that all the SA-β-gal activity we observed could be produced by phagocytic cells, given the occurrence of phagocytosis during development to clear apoptotic cells generated by programmed cell death, and possibly to clear also senescent cells. To estimate the abundance of macrophages, we investigated whether the cells displaying SA-β-gal activity also express the activated macrophage/microglia marker F4/80. As shown in Figure 1D, very few macrophages were found in the medulla oblongata, and while in the spinal cord macrophages clearly showed strong SA-β-gal activity, we detected SA-β-gal activity around cells expressing ISLET1 but not F4/80, indicating that both macrophages and motoneurons have SA-β-gal activity in the developing spinal cord.

### The Cell Cycle Inhibitor p21^CIP1/WAF^ Is Expressed in Endothelial Cells of the Developing Spinal Cord and Some Cells in the Notochord, and p16^INK4A^ Is Expressed in Motoneurons

**Figure 2:**
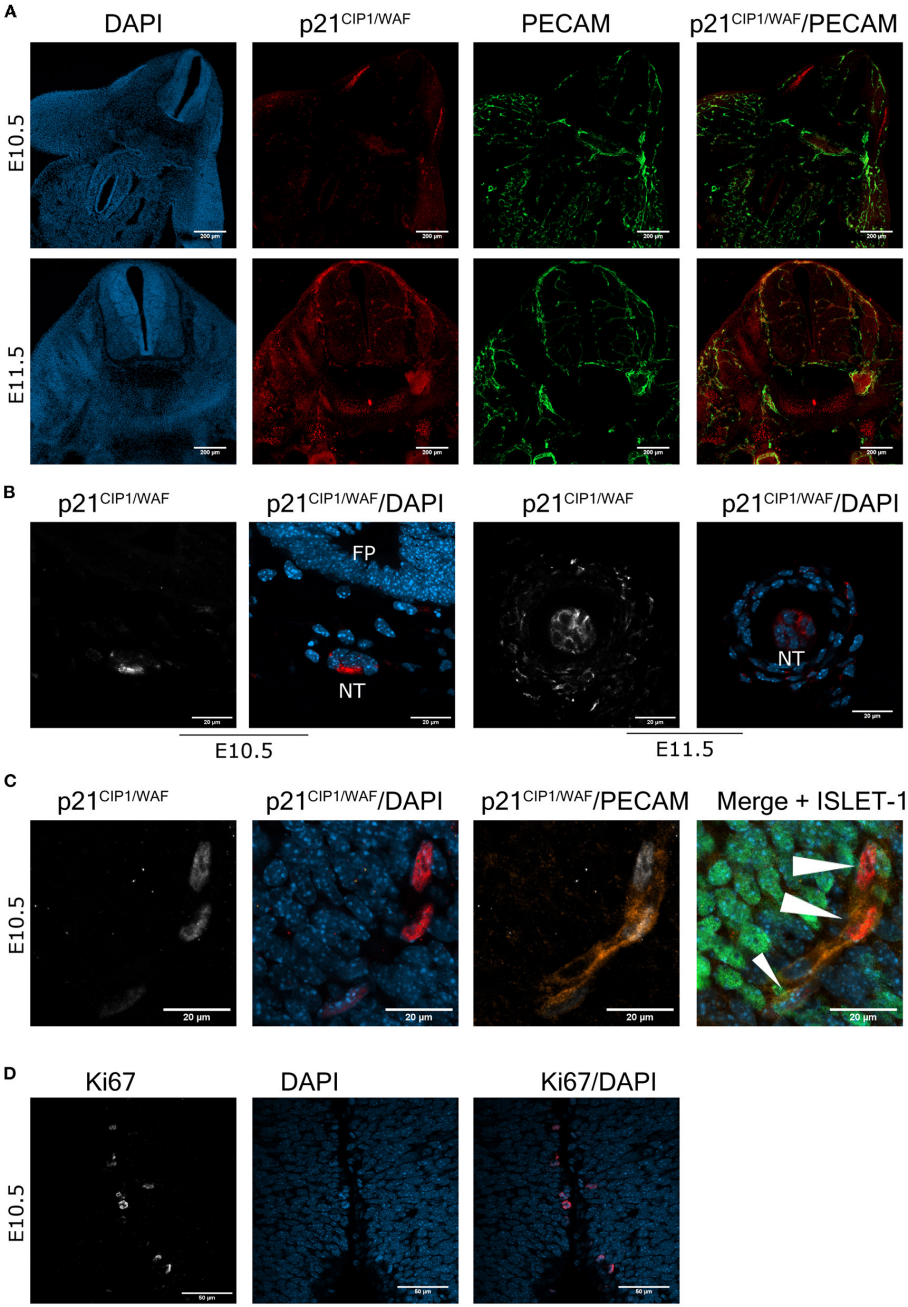
The cell cycle inhibitor p21^CIP1/WAF^ is expressed in the mouse embryonic spinal cord and notochord. **(A)** Endothelial cells express p21^CIP1/WAF^. Brachial or cervical sections from E10.5 and E11.5 embryos, respectively, were processed for immunodetection of p21^CIP1/WAF^ and PECAM. Embryos of the two stages show expression of p21^CIP1/WAF^ in several organs, including the spinal cord. In the spinal cord, p21^CIP1/WAF^ is expressed in endothelial cells labeled with PECAM. Single confocal planes are shown. Scale bars represent 200 μm. **(B)** p21^CIP1/WAF^ is expressed in some notochord cells. Sections of embryos from stages E10.5 and E11.5 were processed as in **(A)**. Notice that p21^CIP1/WAF^ is located in the cytoplasm of notochord (NT) cells, while it is absent in the floor plate (FP) from the E10.5 embryo. Single confocal planes are shown. Scale bars 20 μm. **(C)** p21^CIP1/WAF^ is expressed in PECAM-positive endothelial cells localized in the vessels that surround the motoneuron column, but not in motoneurons themselves in the spinal cord of E10.5 embryos. White arrowheads indicate p21^CIP1/WAF^ -positive nuclei. Single confocal planes are shown. Scale bars 20 μm. **(D)** Only few cells along the central canal of the spinal cord of E10.5 embryos are proliferating, none in the motoneuron zone. Embryos from E10.5 stage were processed for immunofluorescence against Ki67 in cervical sections. Single confocal plane is shown. Scale bars represent 50 μm. Representative images are shown from at least 3 embryos analyzed per immunofluorescence.

To corroborate the senescent phenotype in cells in the embryonic spinal cord and notochord, we analyzed whether the senescence marker p21^CIP1/WAF^ was also expressed. First, we confirmed the specificity of our antibody by analyzing the apical ectodermal ridge, a structure known to express p21^CIP1/WAF^ as part of the developmentally programmed cell senescence (Storer et al., 2013). Accordingly, cells in this structure indeed showed immunoreactivity against this antibody (Supplementary Figure 1A). Then, cross-sections from E10.5 and E11.5 embryos were analyzed for the expression of p21^CIP1/WAF^. Notably, p21^CIP1/WAF^ was expressed in several parts of the embryo, including the spinal cord (Figure 2A) and the notochord, where it was located mainly in the cytoplasm (Figure 2B). We could not find motoneurons with a clear expression of p21^CIP1/WAF^ in the spinal cord (Figure 2C), which was surprising since some of them had high SA-β-gal activity (Figures 1C,D). We identified as endothelial cells (expressing PECAM) the subpopulation of cells in the spinal cord that expressed p21^CIP1/WAF^ (Figure 2C). Endothelial cells in the medulla oblongata and in areas of the forelimb and thorax also expressed p21^CIP1/WAF^ (Supplementary Figures 1B,C). As cellular senescence is characterized by exit from the cell cycle and lack of cell division, we analyzed the expression of the proliferation marker Ki67. We found that only few cells along the central canal of the spinal cord express Ki67, none in the motoneuron zone (Figure 2D).

**Figure 3:**
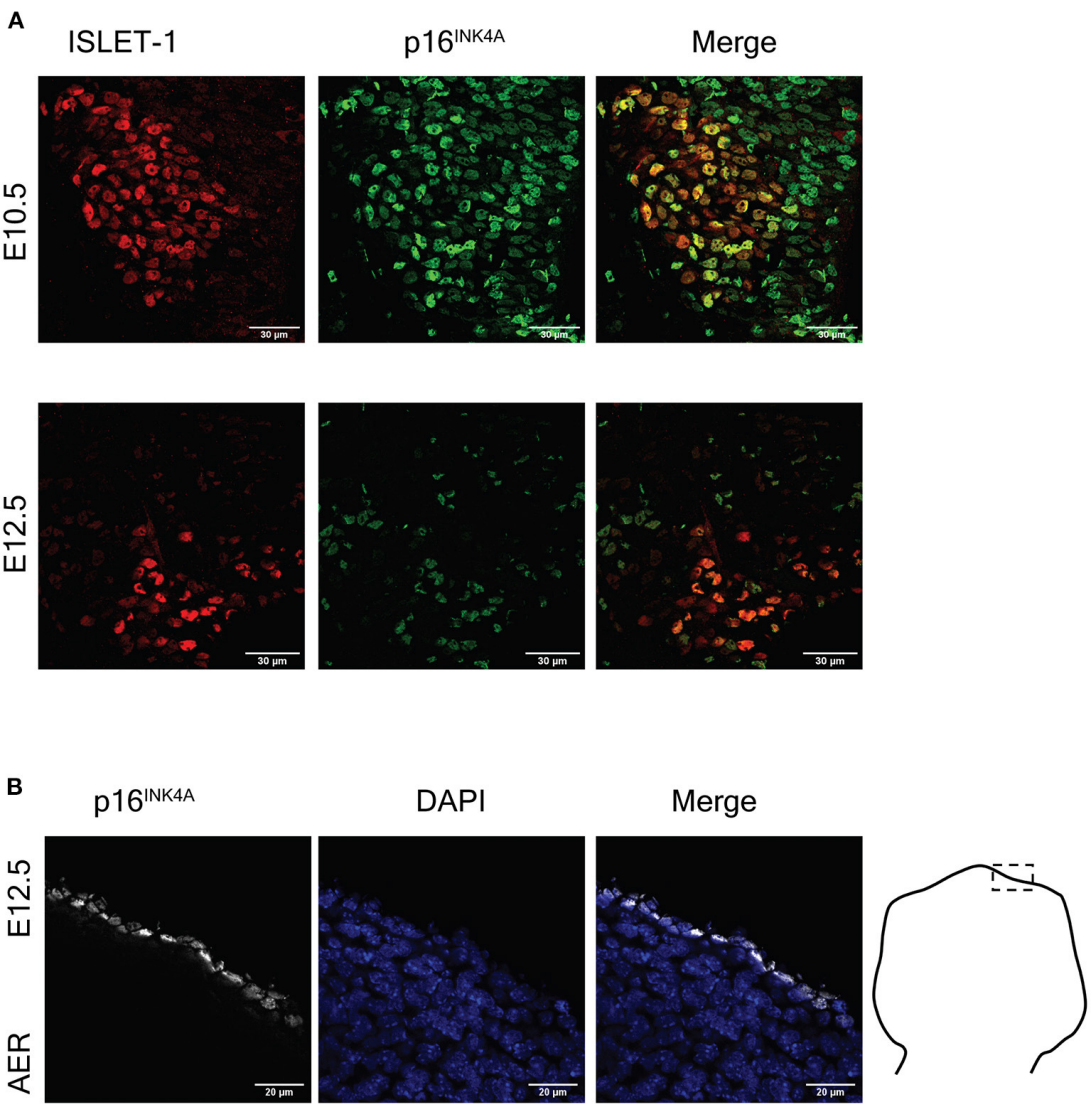
The cell cycle inhibitor p16^INK4A^ is expressed in the mouse embryonic spinal cord and the apical ectodermal ridge. **(A)** Embryonic motoneurons express p16^INK4A^. Representative images of immunofluorescence analysis to detect ISLET-1 and p16^INK4A^ in cervical sections of embryos from stages E10.5 and E12.5 are shown. The number of motoneurons decreases from E10.5 to E12.5 due to programmed cell death. Note that p16^INK4A^ is expressed in abundant cells in this area, several of them also express ISLET-1. At both stages p16^INK4A^ is expressed in several motoneurons. Scale bars 30 μm. **(B)** Cells of the apical ectodermal ridge (AER) express p16^INK4A^. Forelimbs of stage E12.5 were sectioned as indicated in the diagram and processed for immunodetection of p16^INK4A^. Single confocal planes are shown. Scale bars represent 20 μm. Representative images are shown from at least three embryos analyzed per immunofluorescence.

Even though it was reported the lack of expression of p16^INK4A^ during mammalian programmed cell senescence (Munoz-Espin et al., 2013; Storer et al., 2013), senescent cells in the placenta express p16^INK4A^ (Gal et al., 2019), and in the developing zebrafish programmed cell senescence is accompanied by *Cdkn2a* expression (Da Silva-Alvarez et al., 2020). Therefore, we investigated whether motoneurons undergoing programmed cell senescence might express p16^INK4A^ instead of p21^CIP1/WAF^. As shown in Figure 3A, we found abundant cells expressing p16^INK4A^ in the motoneuron zone, and indeed a subpopulation of motoneurons also expressed p16^INK4A^. We verified that the antibody used to detect p16^INK4A^ provide a signal restricted to programmed cell senescence, by immunostaining the limbs at E12.5 (Figure 3B). We observed that only the apical ectodermal ridge immunolabeled positive for p16^INK4A^.

**Figure 4:**
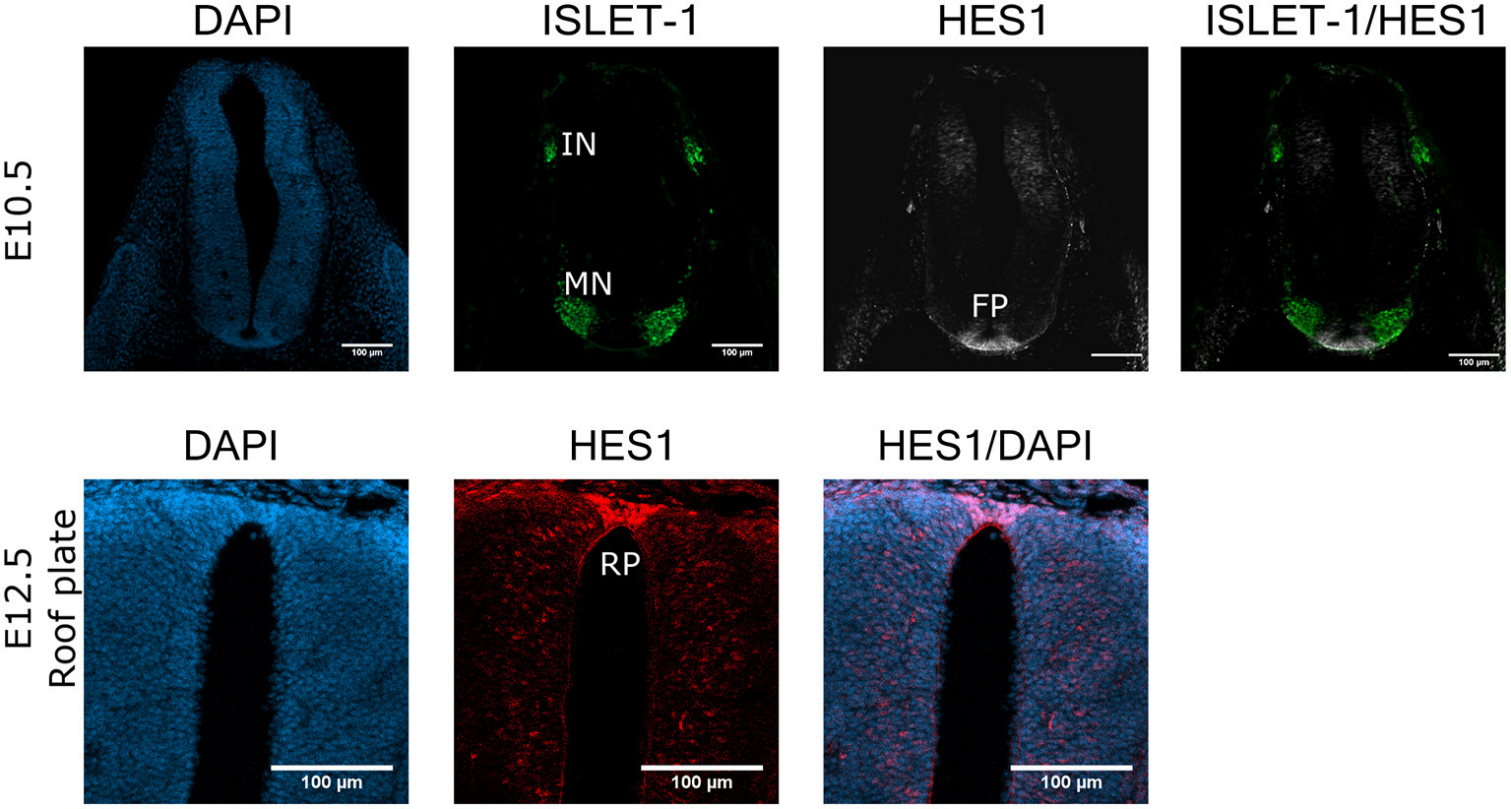
HES1 is expressed in some cells in the floor plate and roof plate in the developing spinal cord. Cross sections of E10.5 or E12.5 embryos (cervical or thoracic, respectively) were analyzed for the expression of HES1 in the spinal cord. The floor plate (FP) of the E10.5 spinal cord contains cells with strong HES1 expression, but none was found in the motoneuron (MN) columns in E10.5 embryos. The roof plate (RP) of the E12.5 embryo also shows clear HES1 expression. Maximal projections of four confocal planes are shown. Scale bars 100 μm. Representative images are shown from at least three embryos anlalyzed.

To distinguish quiescent cells from senescent cells, we analyzed the expression of the transcriptional repressor HES1, which prevents senescence establishment and irreversible cell cycle exit in quiescent cells (Sang et al., 2008; Sueda et al., 2019). Figure 4 shows that motoneurons identified by ISLET-1 expression did not expressed HES1. Interestingly, we found precisely defined domains of HES1 expression in the floor plate of spinal cords at E10.5 (Figure 4, upper panel) and in the roof plate at E12.5 (and Figure 4, lower panel), which lack p21^CIP1/WAF^ expression (Figure 2B, E10.5). The finding that the roof plate expressed HES1 at E12.5, a zone reported to undergo programmed cell senescence (Storer et al., 2013), suggests that in those areas there are both senescent cells and quiescent cells.

### A Subpopulation of Embryonic Motoneurons Are Sensitive to the Senolytic Drug ABT-263

**Figure 5:**
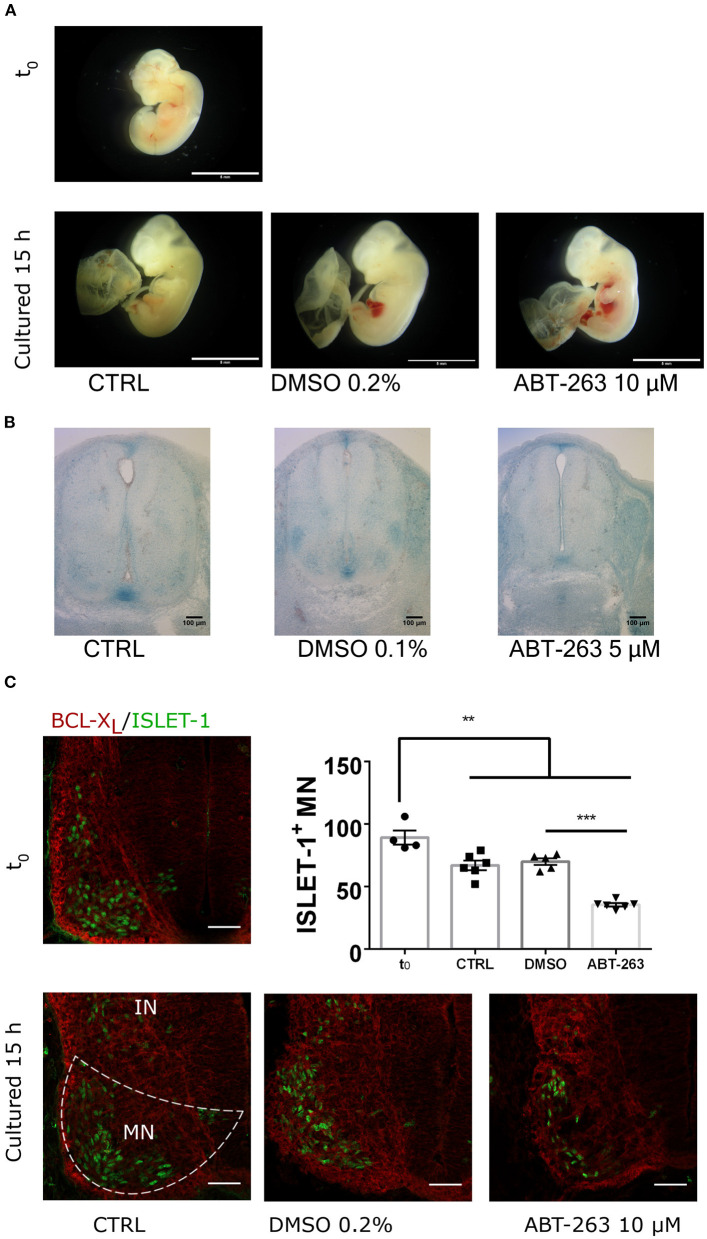
The senolytic ABT-263 reduced SA-β-gal activity in the spinal cord and promotes motoneurons cell death during development. **(A)** Morphology of embryos dissected at E12 (t_0_) and cultured for 15 h, in control medium, supplemented with vehicle only or with 10 μM ABT-263. All culture conditions developed similar growth compared to *in utero* development. Scale bar 5 mm. **(B)** Embryos of the E12 stage were cultured as in **(A)**, stained for SA-β-gal, and cross-sectioned. The treatment with ABT-263 resulted in a decrease of SA-β-gal activity in the motoneuron zone and floor plate. **(C)** Cervical cross-sections of embryos at the beginning (t_0_) or after 15 h cultured as in **(A)** were processed for immunofluorescence to detect BCL-X_L_ and ISLET-1 expression. Single confocal planes are shown. Scale bars 50 μm. Graph plots the quantification of motoneurons (ISLET-1 expressing cells). Each data point represents the number of cells counted in the hemisection of a single embryo. Number of embryos per group: T_0_ = 4, CTRL *n* = 6, DMSO *n* = 5, ABT-263 *n* = 6. Data are plotted as mean ± SEM; significant differences were obtained by one-way ANOVA analysis followed by Bonferroni's multiple comparisons test; **p ≤ 0.01; ***p ≤ 0.001.

**Figure 6:**
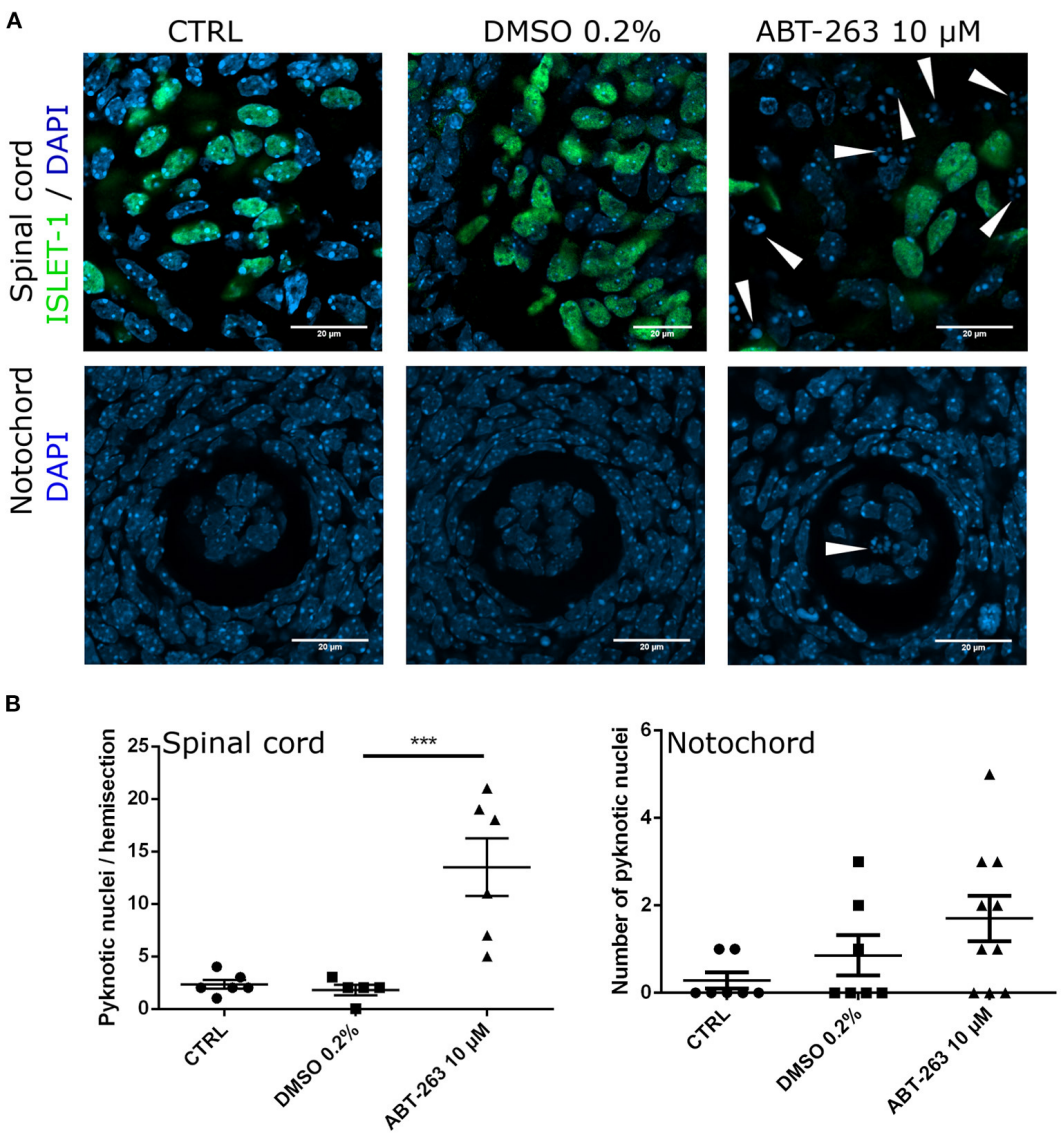
Embryos exposure to the senolytic ABT-263 increased pyknotic nuclei. **(A)** Confocal images of the spinal cord showing the motoneurons and nuclei (upper row), or the notochord (lower row) from embryos cultured with 10 μM ABT-263, vehicle (DMSO 0.2%) or control conditions. **(B)** Left: quantification of pyknotic nuclei in the spinal cord. Each data point represents the number of pyknotic nuclei in the motoneuron zone of a single embryo. At least five embryos were analyzed. Right: quantification of pyknotic nuclei in the notochord. Each data point represents the number of pyknotic nuclei in notochord. Number of embryos per group: CTRL *n* = 7, DMSO *n* = 7, ABT-263 *n* = 10. Data are plotted as mean ± SEM significant differences were obtained from one-way ANOVA followed by Bonferroni's multiple comparisons test. ***p ≤ 0.001.

To confirm whether the subpopulation of motoneurons with detectable SA-β-gal activity, and expressing p16^INK4A^ but not HES1 in the developing spinal cord were indeed senescent, we tested their sensitivity to the senolytic ABT-263. To validate our tools, we compared the toxicity of ABT-263 in proliferative *vs*. senescent mouse embryonic fibroblasts. By quantifying the number of live or dead cells, we confirmed that ABT-263 preferentially kills senescent cells (Supplementary Figure 2). We then collected embryos at stage E12 and cultured them for 15 h to develop them *ex-utero*, either in the presence of ABT-263 or vehicle only. Using serum-free medium, embryos developed similarly to *in vivo*, as shown in Figure 5A and Supplementary Figure 3A. We found that embryos cultured with ABT-263 resulted in decreased activity of SA-β-gal in the spinal cord (Figure 5B), confirming that programmed senescent cells are also susceptible to ABT-263. Since this senolytic acts by inhibiting antiapoptotic BCL-2 family of proteins, we verified the expression of BCL-X_L_ in the motoneuron zone in the developing spinal cord (Figure 5C). To specifically analyze whether ABT-263 promotes motoneuron senescent cells death, the number of ISLET-1 positive cells was quantified in embryos cultured with ABT-263 compared with control or vehicle only conditions. As shown in Figure 5C, exposure to ABT-263 eliminated a subgroup of motoneurons. To verify that the reduction in motoneuron number due to the ABT-263 treatment correlates with increased cell death, pyknotic nuclei were quantified in the motoneuron zone of the spinal cord and notochord. Indeed, ABT-263-treated embryos showed a higher amount of pyknotic nuclei in the motoneuron zone (Figure 6A, upper row and Figure 6B, left). The number of pyknotic nuclei in the notochord also showed a trend to increase (Figure 6A, lower row and Figure 6B, right). All together, the data shown suggest that a subpopulation of motoneurons undergo programmed cell senescence, perhaps contributing to eliminate supernumerary motoneurons.

Previous observations have shown that in addition to apoptosis other cellular processes might contribute to the programmed cell death of motoneurons, and cell death by autophagy was proposed (Sanchez-Carbente et al., 2005). To investigate whether autophagy signaling contributes to motoneurons cell death, embryos collected at stage E12 were cultured 15 h in the presence of the autophagy inhibitor spautin-1 (Liu et al., 2011) or vehicle only. Again, embryos cultured in serum-free medium reproduced *in vivo* development (Supplementary Figure 3A); autophagy inhibition was corroborated by Western blot detection of decreased LC3-II levels (Supplementary Figure 3B). As occurs during normal development, the number of motoneurons decreased during the course of the culture, reproducing the *in vivo* developmental window of motoneurons programmed cell death. We found that pharmacological inhibition of autophagy did not prevent motoneurons programmed cell death (Supplementary Figure 3C). This result suggests that autophagic cell death does not contribute to establish the final number of motoneurons during spinal cord development. In summary, we observed that embryonic motoneurons are insensitive to autophagy inhibition, but sensitive to the senolytic ABT-263, suggesting that programmed cell senescence contributes to the adjustment of motoneurons number. It would be necessary to prevent motoneuron senescence establishment to test this idea.

## Discussion

**Figure 7:**
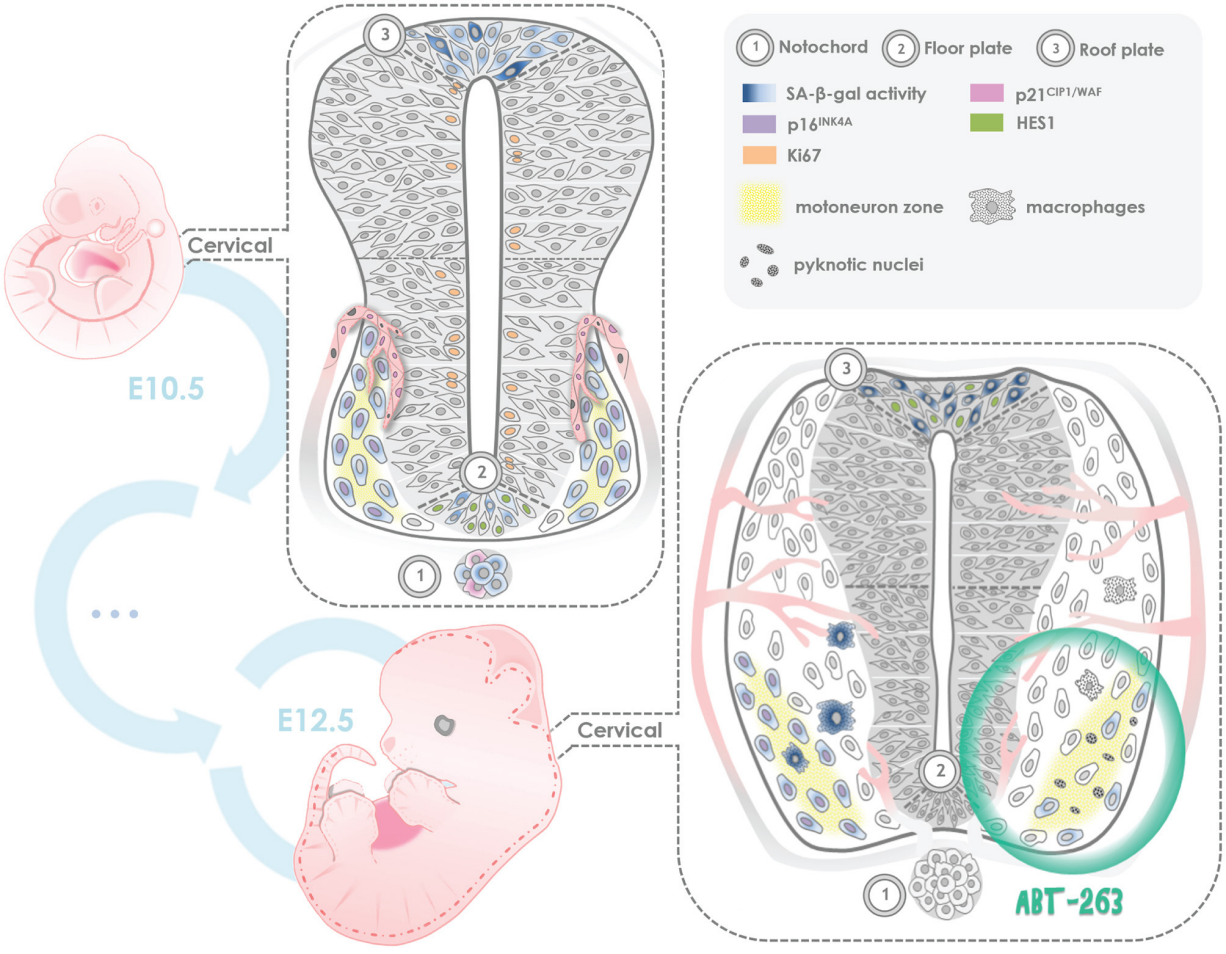
Summary of programmed cell senescence markers found in the mouse embryonic spinal cord and notochord. SA-beta-gal activity and expression of p21^CIP1/WAF^ were detected since stage E10.5 and through stage E12.5 in endothelial cells of blood vessels; p16^INK4A^ expression was found in motoneurons, with lack of expression of Ki67, a proliferation marker, and lack of HES1 expression, a quiescence maintenance factor. Notably, in the floor plate and roof plate both SA-β-gal activity and HES1 expression were observed. We propose that in those regions there are both senescent cells and quiescent cells. In some notochord cells, SA-β-gal activity and cytosolic expression of p21^CIP1/WAF^ were found. Given that the notochord is a signaling center, perhaps the few senescent cells we observed contribute to the secretion of development factors. In *ex-utero* development of E12 embryos, a subpopulation of motoneurons resulted sensitive to the senolytic ABT-263. We propose that programmed cellular senescence cooperates with apoptosis to adjust the number of motoneurons during spinal cord development, and the transient presence of senescent cells contributes to instruct the differentiation of neuronal populations and endothelial cells.

The identification of programmed cell senescence as a developmental process in amphibians, salamanders, fish, birds and mammals, supports the notion that the senescent program emerged during the evolution of vertebrates, and its mechanism is conserved. It is fundamental to uncover the role of programmed cell senescence during development, as well as to decipher the molecular basis for cellular senescence induction under developmental cues. As summarized in Figure 7, in this work we found that several cell types acquired senescent features in the spinal cord and notochord of embryos at developmental stages E10.5 - E12.5.

It is important to consider that the finding of endothelial cells of blood vessels with SA-β-gal activity and p21^CIP1/WAF^ expression needs cautious interpretation. Unlike the programmed cell senescence that occurs in transient structures that are eliminated during development, endothelial cells remain in the blood vessels, where they become fundamental components. Consistent with our data, previous studies have shown that during endothelial cell differentiation two markers of senescence are observed: p21^CIP1/WAF^, that mediates the cell cycle arrest necessary for endothelial cell differentiation (Zeng et al., 2006; Marcelo et al., 2013), and SA-β-gal activity, that occurs during differentiation of human prostate epithelial cells (Untergasser et al., 2003). A report that elegantly linked the concepts of senescence and differentiation was published recently (Li et al., 2018). By using genetic lineage tracing, Li and colleagues showed that in the apical ectodermal ridge at mid-stage (E10.5–E13.5) there is a population of p21^CIP1/WAF^ -positive and SA-β-gal-positive cells (i.e., senescent cells). Interestingly, by late-stage (E15.5–E16.5) or after birth, a subset of the previously senescent cells re-entered the cell cycle, proliferated, exhibited epithelial fate and contributed to tissues (Li et al., 2018). Thus, endothelial cells that express p21^CIP1/WAF^ in the developing spinal cord may engage in a senescent program and nevertheless form the blood vessels later in development.

We found some cells in the notochord with SA-beta-gal activity, expression of p21^CIP1/WAF^, and possibly susceptible to the senolitic ABT-263 (Figure 6), supporting the senescent nature of those few notochord cells. Given that the notochord is a signaling center for the differentiation of neuron populations of the spinal cord, perhaps the few senescent cells we observed contribute to the secretion of developmental factors. We noticed that p21^CIP1/WAF^ was found in the cytoplasm of notochord cells. While p21^CIP1/WAF^ inhibits cell cycle when it is located in the nucleus, in the cytoplasm it has an anti-apoptotic function due to its ability to bind and inhibit the activity of proapoptotic proteins (Karimian et al., 2016).

A contrasting observation was to find in the floor plate and roof plate both SA-β -gal activity and HES1 (a quiescence maintenance factor) expression. One possibility is that in those regions there are both senescent cells and quiescent cells. Nevertheless, SA-β-gal activity could also occur in quiescent cells expressing HES1 (Baek et al., 2006). To determine whether indeed the same cell expressing HES1 had high SA-β-gal, we attempted to detect both HES1 and SA-β-gal in the same preparations, but it resulted in strong background and nonspecific binding of the HES1 antibody, probably caused by the fixation protocol for SA-β-gal. Other reports also show that SA-β-gal is detectable in non-senescent cells such as the yolk sac, visceral endoderm, and macrophages (Cristofalo, 2005; Yang and Hu, 2005; Huang and Rivera-Perez, 2014; Hall et al., 2017). The activation of SA-β-gal is also caused by cellular differentiation induced by TGF-β (Untergasser et al., 2003). Nevertheless, previous articles showed SA-β-gal in the neural tube, and concluded that programmed senescence occurs in the roof plate of the spinal cord (Munoz-Espin et al., 2013; Storer et al., 2013; Czarkwiani and Yun, 2018). Indeed, p21^CIP1/WAF^ -positive cells were detected in the closing neural tube (Storer et al., 2013), but expression of p21^CIP1/WAF^ or other cell cycle inhibitor was not shown in the roof plate. Thus, we consider open the possibility that in addition to senescence, quiescence can be accompanied by SA-β-gal in the roof plate.

Given the transient nature of programmed cell senescence, as they are eliminated by macrophages (Storer et al., 2013), cellular senescence could contribute to eliminate unwanted cells in concert with programmed cell death. This presumed function is supported by finding senescent cells during organ regression (i.e., the mesonephros), and in regions and developmental windows where cell population is balanced (i.e., the endolymphatic sac of the inner ear) (Da Silva-Álvarez et al., 2019). In this work, we observed that a subpopulation of motoneurons undergo programmed cell senescence, revealed by SA-β-gal activity, p16^INK4A^ expression, lack of Ki67 and HES1 expression, and susceptibility to the senolytic ABT-263. Interestingly, senescent motoneurons appear at a developmental window in which supernumerary motoneurons are being eliminated. We propose that programmed cell senescence cooperates with apoptosis to adjust the number of motoneurons during spinal cord development, either by secreting differentiation factors or signals to be cleared by macrophages. In a similar way that senescent cells in the apical ectodermal ridge reintegrated into the tissue (Li et al., 2018), it could also occur that a subpopulation of senescent motoneurons continues to fully differentiate into postmitotic neurons and remain integrated in the tissue.

To confirm a developmental role of senescent motoneurons, we should be able to prevent the acquisition of the senescent phenotype and observe a disruption of the developmental pattern of the neural tube, or an increase in the total number of motoneurons. Currently this experiment is not possible, since TGF-β is the only signaling pathway described to induce programmed cell senescence (Munoz-Espin et al., 2013) but it has also a crucial role in neural tube development. So, if we inhibit TGF-β pathway we won't be able to distinguish the developmental role of TGF-β from the role of senescent cells *per se*. The fact that we detected p16^INK4A^ expression (but not p21^CIP1/WAF^) in some motoneurons, opens the possibility to analyze whether in the absence of p16^INK4A^ motoneurons senescence could be prevented. Cellular senescence mediated by p16^INK4A^ has also been observed during placenta development (Gal et al., 2019), and *Cdkn2a* mRNA was detected in programmed cell senescence in zebrafish (Da Silva-Alvarez et al., 2020); it is possible, then, that different cell types undergoing programmed cell senescence vary in the tumor suppressor gene they expressed.

In summary, our findings allow us to speculate that (1) SA-β-gal activation may occur during the quiescent phenotype of the floor and roof plates, (2) endothelial cells are probably engaged in a phenomenon of reversible senescence, (3) a subpopulation of notochord cells become senescent and could secrete morphogens, and (4) motoneuron programmed senescence could contribute to the elimination of motoneurons during spinal cord development. Further experiments will be needed to test these hypotheses.

## Data Availability Statement

The original contributions presented in the study are included in the article/Supplementary Material, further inquiries can be directed to the corresponding author/s.

## Ethics Statement

The animal study was reviewed and approved by Internal Committee of Care and Use of Laboratory Animals of the Institute of Cell Physiology- UNAM (IFC-SCO51-18).

## Author Contributions

JD-B and SC-O contributed to conception and designed of the study. JD-B performed most of the experiments and wrote the first draft of the manuscript. PA-R performed experiments, acquired data and prepared Figures 3, 7. All authors contributed to manuscripts revision, read and approved the submitted version of the manuscript, and are accountable for the content of the work.

## Conflict of Interest

The authors declare that the research was conducted in the absence of any commercial or financial relationships that could be construed as a potential conflict of interest.

## Supplementary Material

Supplementary Figure 1 The cell cycle inhibitor p21^CIP1/WAF^ is expressed in endothelial cells of the medulla oblongata and forelimb. **(A)** The specificity of the p21^CIP1/WAF^ antibody was validated by analyzing the expression of p21^CIP1/WAF^ in the apical ectodermal ridge of a E12.5 embryo. The section area is indicated on the diagram on the right. Single confocal planes are shown. Scale bars represent 20 μm. **(B)** Cross-section analysis of the medulla oblongata from a representative E12 embryo as indicated on the right. Confocal microscopy shows that p21^CIP1/WAF^ is expressed in endothelial (PECAM-positive) cells. In the upper panel it is shown a low magnification, in the lower panel a higher magnification is shown. Single confocal planes are shown. Scale bars represent 100 or 20 μm. **(C)** Expression of p21^CIP1/WAF^ in endothelial cells of the forelimb and thorax of a representative E13.5 embryo. Single confocal planes are shown. Scale bars 100 μm.

Supplementary Figure 2 *In vitro* validation of the senolytic effect of ABT-263. Proliferative (non-senescent) and senescent MEFs were treated for 20 h with ABT-263 at the indicated concentrations and the cytotoxic effect was determined quantifying live and dead cells. Since 5 μM ABT-263 induced senescent fibroblasts death concomitant with a reduction of cells alive. We noticed a decrease in the number of live non-senescent MEFs with 10 μM ABT-263, although the number of dead cells was not affected. Each group was cultured in triplicate wells, and five random fields in each well were used to count the number of live or dead cells. Data are plotted as mean ± SEM significant differences were obtained from one-way ANOVA followed by Bonferroni's multiple comparisons test; **p* ≤ 0.05, ***p* ≤ 0.01.

Supplementary Figure 3 Pharmacological inhibition of autophagy does not alter the amount of motoneurons. **(A)** Morphology of embryos dissected at E12 (t_0_) and cultured for 15 h, in control medium, supplemented with vehicle only or with 10 μM spautin-1. All culture conditions developed similar growth compared to *in utero* development. Scale bar 5 mm. Graph shows the weight gain of the embryos after culture. Each dot represents an embryo **(B)** Treatment with spautin-1 inhibits autophagy, as it results in a decrease of LC3-II levels compared to control (CTRL) or vehicle (DMSO). Western blot to detect indicated proteins from total lysates obtained from embryonic spinal cords of E12 embryos cultured with spautin-1, vehicle or control for 15 h. As a reference to distinguish LC3-II, a lysate from mouse embryonic fibroblasts (MEFs) cultured with chloroquine (CQ) was also loaded. Graph shows densitometric LC3-II levels normalized with β-actin as loading control. Data are plotted as mean ± SEM, significant differences were obtained from one-way ANOVA followed by Bonferroni's multiple comparisons test; **p* ≤ 0.05. *n* = 4. **(C)** ISLET-1 positive motoneurons were quantified in cervical cross-sections from embryos dissected at E12 and developed *ex-utero* for 15 h with indicated treatments. The results of four independent embryo cultures are shown, with at least two embryos per condition per culture. Each data point represents the number of cells quantified in the hemisection of a single embryo. The number of motoneurons significantly decreased in all conditions after culture, as occurs during this stage of embryo development, but pharmacologic inhibition of autophagy with spautin-1 did not affect the amount of surviving motoneurons. Data are plotted as mean ± SEM, significant differences were obtained from one-way ANOVA followed by Bonferroni's multiple comparisons test; **p* ≤ 0.05.

## Abbreviations

ANOVA: Analysis of Variance
BCL-X_L_: B-cell lymphoma-extra large
p21^CIP1/WAF^: Cyclin-dependent kinase inhibitor 1A
CDKN1B/p27: Cyclin-dependent kinase inhibitor 1B
p16^INK4A^: Cyclin-dependent kinase inhibitor 2A
CDKN2B/p15: Cyclin-dependent kinase inhibitor 2B
CQ: Chloroquine
CTRL: Control
DMEM: Dulbecco's Modified Eagle Medium
DMEM/F12: Dulbecco's Modified Eagle Medium/Nutrient Mixture F-12
DMSO: Dimethyl Sulfoxide
ERK1/2: Extracellular signal–Regulated Kinases 1 and 2
FP: Floor plate
IN: Interneurons
LC3: Microtubule-associated protein 1A/1B-light chain 3
MEFs: Mouse Embryonic Fibroblasts
MN: Motoneurons
PBS: Phosphate-Buffered Saline
PECAM: Platelet Endothelial Cell Adhesion Molecule
PI3K/FOXO: Phosphoinositide-3-Kinase/Forkhead box O
RP: Roof plate
SA-β-gal: Senescence-associated beta-galactosidase
SDS: Sodium Dodecyl Sulfate
SEM: Standard Error of the Mean
TGF-β: Transforming Growth Factor beta.
