# Supplementary figures and images for: Programmed Cell Senescence in the Mouse Developing Spinal Cord and Notochord
Source: Front Cell Dev Biol. 2021 Jan 26;9:587096. doi: 10.3389/fcell.2021.587096 (PMC7870793; doi:10.3389/fcell.2021.587096)

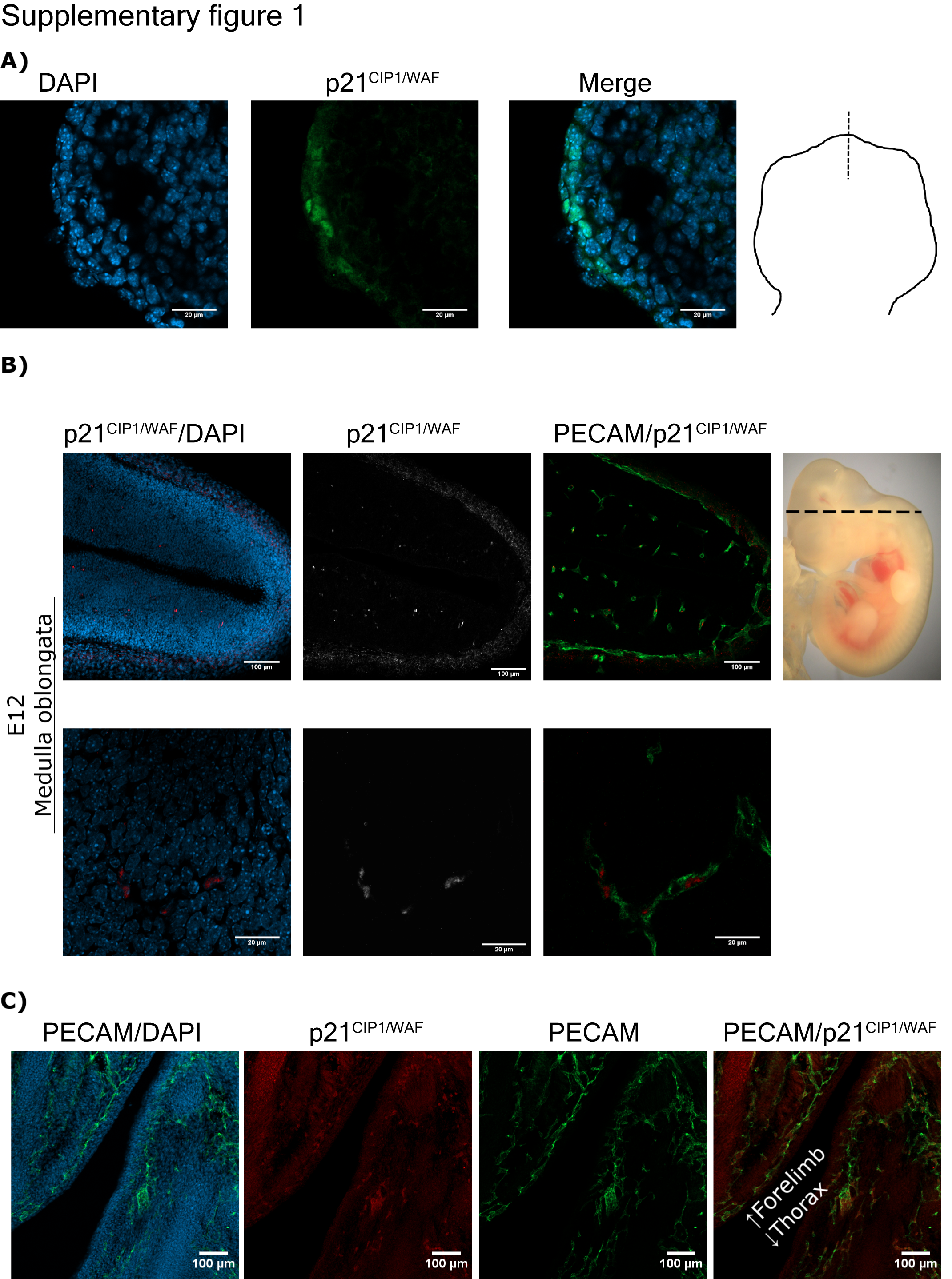

Supplement: Supplementary Figure 1 — The cell cycle inhibitor p21CIP1/WAF is expressed in endothelial cells of the medulla oblongata and forelimb. (A) The specificity of the p21CIP1/WAF antibody was validated by analyzing the expression of p21CIP1/WAF in the apical ectodermal ridge of a E12.5 embryo. The section area is indicated on the diagram on the right. Single confocal planes are shown. Scale bars represent 20 μm. (B) Cross-section analysis of the medulla oblongata from a representative E12 embryo as indicated on the right. Confocal microscopy shows that p21CIP1/WAF is expressed in endothelial (PECAM-positive) cells. In the upper panel it is shown a low magnification, in the lower panel a higher magnification is shown. Single confocal planes are shown. Scale bars represent 100 or 20 μm. (C) Expression of p21CIP1/WAF in endothelial cells of the forelimb and thorax of a representative E13.5 embryo. Single confocal planes are shown. Scale bars 100 μm. [file Image_1.TIF]

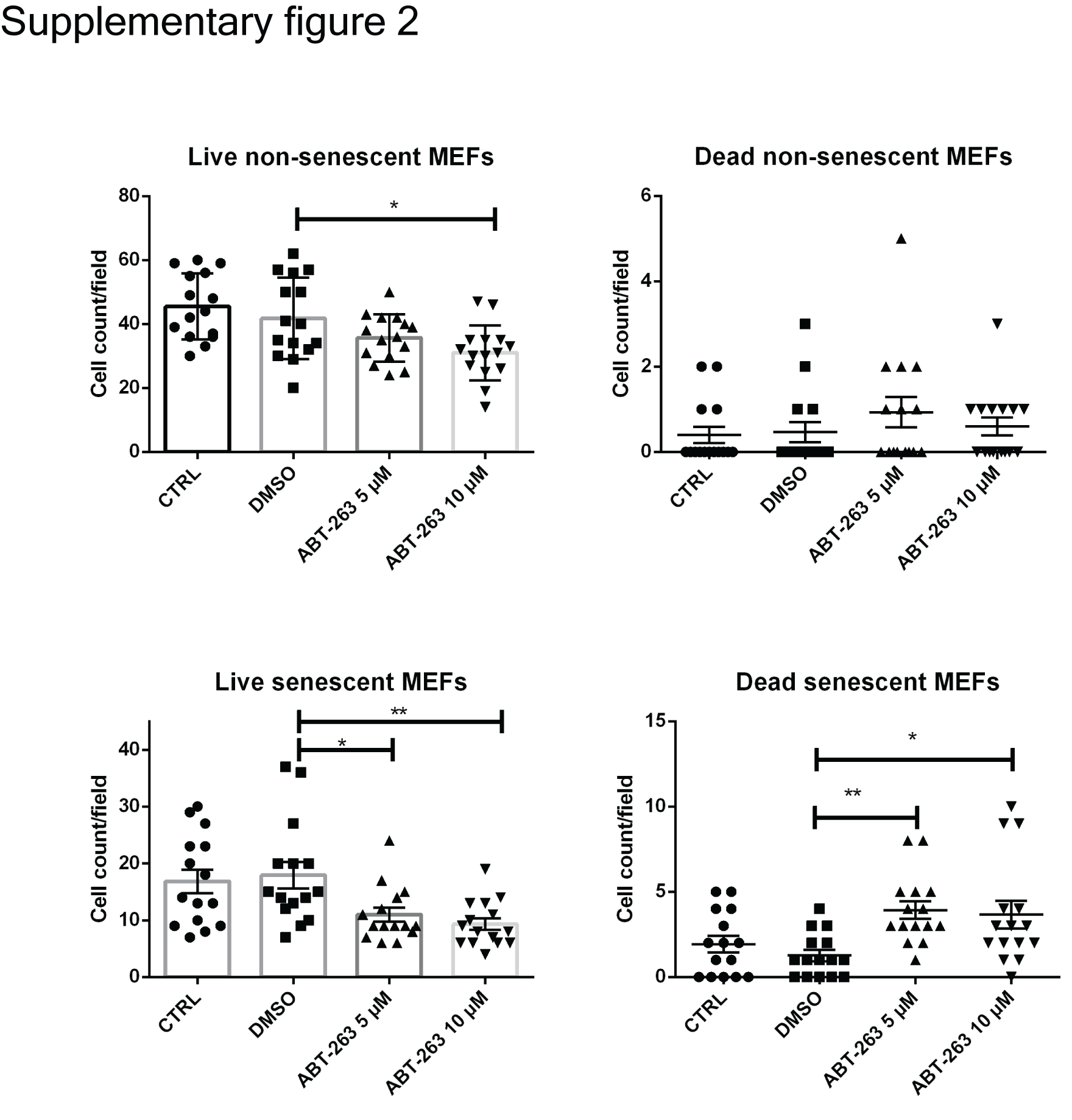

Supplement: Supplementary Figure 2 — In vitro validation of the senolytic effect of ABT-263. Proliferative (non-senescent) and senescent MEFs were treated for 20 h with ABT-263 at the indicated concentrations and the cytotoxic effect was determined quantifying live and dead cells. Since 5 μM ABT-263 induced senescent fibroblasts death concomitant with a reduction of cells alive. We noticed a decrease in the number of live non-senescent MEFs with 10 μM ABT-263, although the number of dead cells was not affected. Each group was cultured in triplicate wells, and five random fields in each well were used to count the number of live or dead cells. Data are plotted as mean ± SEM significant differences were obtained from one-way ANOVA followed by Bonferroni's multiple comparisons test; *p ≤ 0.05, **p ≤ 0.01. [file Image_2.TIF]

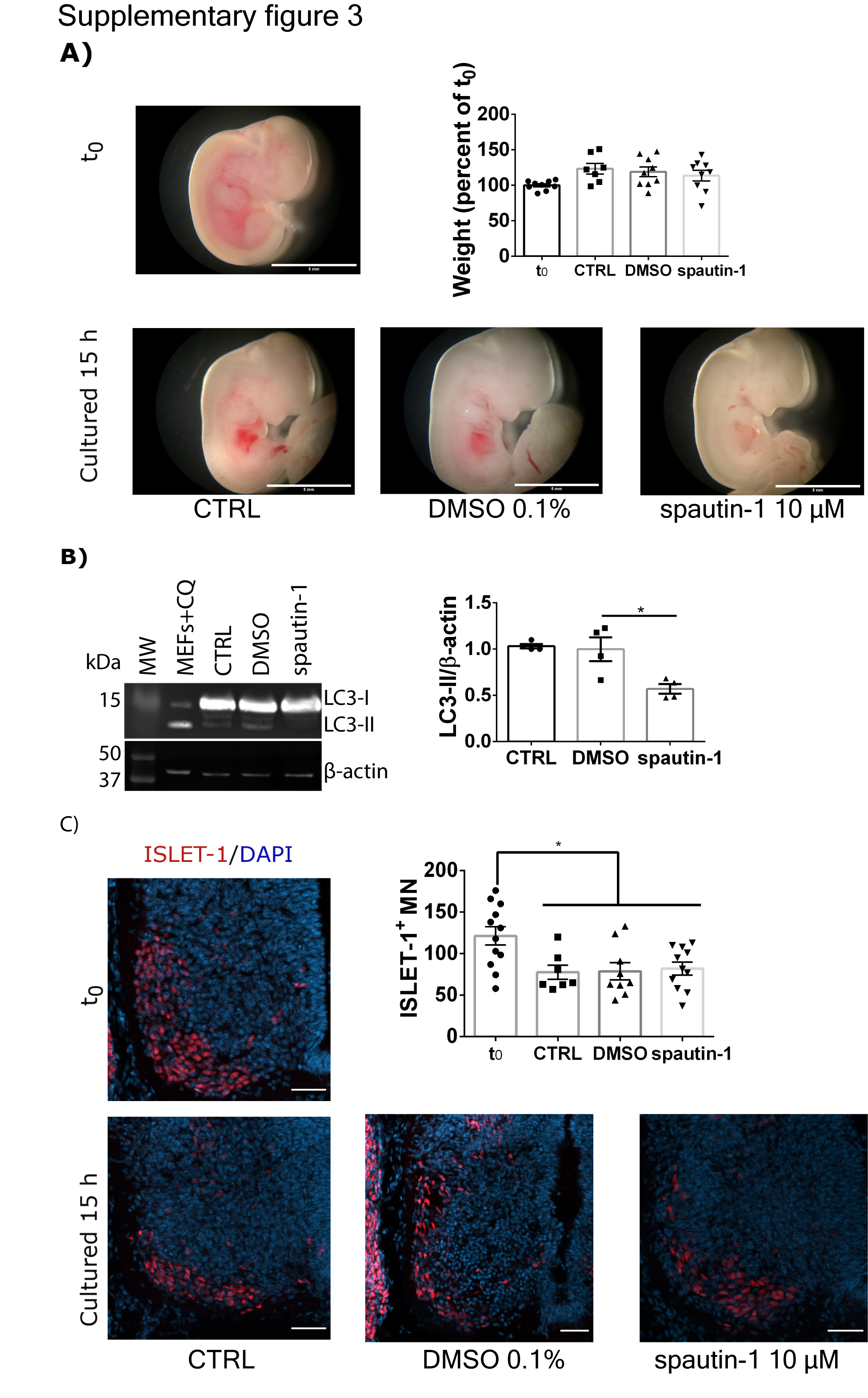

Supplement: Supplementary Figure 3 — Pharmacological inhibition of autophagy does not alter the amount of motoneurons. (A) Morphology of embryos dissected at E12 (t0) and cultured for 15 h, in control medium, supplemented with vehicle only or with 10 μM spautin-1. All culture conditions developed similar growth compared to in utero development. Scale bar 5 mm. Graph shows the weight gain of the embryos after culture. Each dot represents an embryo (B) Treatment with spautin-1 inhibits autophagy, as it results in a decrease of LC3-II levels compared to control (CTRL) or vehicle (DMSO). Western blot to detect indicated proteins from total lysates obtained from embryonic spinal cords of E12 embryos cultured with spautin-1, vehicle or control for 15 h. As a reference to distinguish LC3-II, a lysate from mouse embryonic fibroblasts (MEFs) cultured with chloroquine (CQ) was also loaded. Graph shows densitometric LC3-II levels normalized with β-actin as loading control. Data are plotted as mean ± SEM, significant differences were obtained from one-way ANOVA followed by Bonferroni's multiple comparisons test; *p ≤ 0.05. n = 4. (C) ISLET-1 positive motoneurons were quantified in cervical cross-sections from embryos dissected at E12 and developed ex-utero for 15 h with indicated treatments. The results of four independent embryo cultures are shown, with at least two embryos per condition per culture. Each data point represents the number of cells quantified in the hemisection of a single embryo. The number of motoneurons significantly decreased in all conditions after culture, as occurs during this stage of embryo development, but pharmacologic inhibition of autophagy with spautin-1 did not affect the amount of surviving motoneurons. Data are plotted as mean ± SEM, significant differences were obtained from one-way ANOVA followed by Bonferroni's multiple comparisons test; *p ≤ 0.05. [file Image_3.TIF]
